# Complete Genome Sequence of the Methanogen *Methanoculleus bourgensis* BA1 Isolated from a Biogas Reactor

**DOI:** 10.1128/genomeA.00568-16

**Published:** 2016-06-23

**Authors:** Irena Maus, Daniel Wibberg, Anika Winkler, Alfred Pühler, Anna Schnürer, Andreas Schlüter

**Affiliations:** a Center for Biotechnology, Bielefeld University (CeBiTec), Institute for Genome Research and Systems Biology, Bielefeld, Germany; b Department of Microbiology, Biocenter, Swedish University of Agricultural Sciences, Uppsala, Sweden

## Abstract

*Methanoculleus bourgensis* BA1, a hydrogenotrophic methanogen*,* was isolated from a laboratory-scale biogas reactor operating under an elevated ammonium concentration. Here, the complete genome sequence of *M. bourgensis* BA1 is reported. The availability of the BA1 genome sequence enables detailed comparative analyses involving other *Methanoculleus* spp. representing important members of microbial biogas communities.

## GENOME ANNOUNCEMENT

Frequently, members of the genus *Methanoculleus* were described as playing an important role in different biogas reactor systems (1, 2). In particular, the species *Methanoculleus bourgensis* was found to be dominant in several biogas systems. Moreover, different studies described the prevalence of *M. bourgensis* in reactors performing syntrophic acetate oxidation (SAO) under high ammonium concentrations (3–5), indicating the importance of this methanogen in corresponding communities. Isolation and/or cocultivation of *M. bourgensis*, together with acetate-oxidizing bacteria (4, 6) such as *Clostridium ultunense* (7), led to the assumption that syntrophic association may play an important role for members of the genus *Methanoculleus*. Bioaugmentation involving *Methanoculleus* spp. in coculture with SAO bacteria was discussed as a feasible approach to shorten the adaptation period of digesters operating under high ammonium/ammonia concentrations (3, 8).

The objective of this work was to sequence the methanogen *M. bourgensis* BA1 (9) originating from a Swedish lab-scale continuous stirred tank reactor (37°C) operating under an elevated ammonium concentration (6.4 g 1^−1^ NH_4_+ N) and utilizing alfalfa silage for methane production. Furthermore, the availability of the *M. bourgensis* BA1 genome sequence and insights into its predicted metabolic capabilities provide reference points for comparative analyses comprising other methanogenic species of *Archaea* from biogas communities.

Strain BA1 was isolated as described previously (9, 10). The 16S rRNA gene sequence analysis classified the isolate as a member of the species *M. bourgensis* with 99% sequence identity to the 16S rRNA gene of strain MS2^T^ (11). Genomic DNA of strain BA1 was isolated using the Qiagen blood and tissue kit and sequenced applying the paired-end protocol on an Illumina MiSeq system. The 2,155,212 reads obtained, accounting for 565,780,211 bp of sequence information, were *de novo* assembled using the GS *de novo* assembler version 2.8 software. The assembly resulted in 14 scaffolds comprising 48 contigs. An *in silico* gap closure approach (12) was applied to close all gaps between contigs and circularize the genome. The complete BA1 chromosome has a size of 2,551,189 bp, featuring a GC content of 60.89%. Annotation of the genome sequence was performed within the annotation system GenDB version 2.0 (13) and resulted in the detection of 2,528 protein-coding sequences, 45 tRNA genes, and one *rrn* operon.

Interpretation of the *M. bourgensis* BA1 genome sequence revealed that all genes required for hydrogenotrophic methanogenesis were identified. Moreover, genes encoding a formate transporter (*fdhC*) and a formate dehydrogenase operon (*fdhA*-*B*) for growth on formate as an alternative methanogenic substrate were found. Since strain BA1 was isolated from a habitat rich in ammonium/ammonia, genes involved in nitrogen metabolism were analyzed. Similar to the type strain *M. bourgensis* MS2^T^ (11, 14), the BA1 genome encodes neither a methylammonium permease nor the putative archaeal ammonium uptake system Amt predicted to transport NH_4_+. The missing ammonium transporter may indicate an adaptation of the strain to environments rich in ammonium/ammonia. Furthermore, strain BA1 harbors genes encoding different potassium transporters and a glycine betaine/proline transport system that may contribute to compatible solute accumulation as response to high osmolarity.

### Nucleotide sequence accession number.

This whole-genome shotgun project has been deposited in the EMBL/GenBank database (EBI, NCBI) under the accession number LT549891 (Study ID: PRJEB13327).
